# Subjektive Sicherheit zur Steigerung der Akzeptanz des automatisierten und vernetzten Fahrens

**DOI:** 10.1007/s10010-021-00500-y

**Published:** 2021-07-02

**Authors:** Uwe Drewitz, Marc Wilbrink, Michael Oehl, Meike Jipp, Klas Ihme

**Affiliations:** Institut für Verkehrssystemtechnik, Deutsches Zentrum für Luft- und Raumfahrt e. V. (DLR), Lilienthalplatz 7, 38108 Braunschweig, Deutschland

## Abstract

Ein wichtiger Faktor für die Akzeptanz und damit die Verbreitung automatisierten und vernetzten Fahrens (AVF) ist der Grad der subjektiven Unsicherheit (Ungewissheit), den Nutzende bei der Interaktion mit automatisierten Fahrzeugen empfinden. Subjektive Unsicherheiten treten immer dann auf, wenn Personen aufgrund fehlender Erfahrung oder fehlender Informationen nicht in der Lage sind, den weiteren Verlauf einer Situation oder zukünftige Ereignisse vorherzusagen. Treten bei der Nutzung automatisierter Fahrzeuge solche Unsicherheiten auf, wird die Herausbildung von Vertrauen und damit von Akzeptanz für diese Technologie durch die Unsicherheit begleitende negative Emotionen beeinträchtigt. Im Rahmen des Projekts AutoAkzept (Automatisierung ohne Unsicherheit zur Erhöhung der Akzeptanz Automatisierten und Vernetzten Fahrens) wurden Lösungen für nutzerfokussierte Automatisierung entwickelt, die Fahrzeuginsassen in den Mittelpunkt der Systementwicklung stellen. Nutzerfokussierte Systeme berücksichtigen in der Mensch-Maschine-Interaktion zwei grundlegende menschliche Bedürfnisse, das Bedürfnis, zu verstehen (*need to understand)* und das Bedürfnis, verstanden zu werden (*need to be understood*). Dazu setzen nutzerfokussierte Systeme verschiedene Sensoren ein, um subjektive Unsicherheiten und ihre Einflussfaktoren in Echtzeit zu erkennen, diese Informationen mit Kontextdaten zu integrieren und Anpassungen vorzunehmen, die subjektive Unsicherheiten reduzieren. Die systemischen Anpassungen nutzerfokussierter Systeme folgen dabei einem ganzheitlichen Ansatz und berücksichtigen die Ebenen der Fahrzeugführung, der Interieuranpassung und Informationsdarbietung sowie der Zielführung. Durch die Reduzierung oder Vermeidung subjektiver Unsicherheiten unterstützen die Entwicklungen des Projekts eine positive, komfortable Benutzererfahrung und tragen zur Erhöhung der Akzeptanz von AVF bei. Die Arbeit präsentiert hierzu Forschungsergebnisse von AutoAkzept zu den Themen Zustands- und Aktivitätsmodellierung von Nutzenden sowie bedarfsgerechte Adaptionsstrategien, die einzelne Lösungsbausteine für die Umsetzung nutzerfokussierter Automation bilden.

## Abstract (en)

An important factor for the acceptance and thus the spread of automated and connected driving (ACD) is the degree of subjective uncertainty that users experience when interacting with automated vehicles. Subjective uncertainties always occur when people are not able to predict the further course of a situation or future events due to lack of experience or information. If such uncertainties occur during the use of automated vehicles, the development of trust and thus acceptance of this technology is impaired by the negative emotions accompanying subjective uncertainties. Within the AutoAkzept project (which full title translates to: Automation without uncertainty to increase the acceptance of automated and connected driving), solutions for user-focused automation have been developed that put vehicle occupants at the center of system development. User-focused systems take into account two basic human needs in human-machine interaction, the need to understand and the need to be understood. For this purpose, user-focused systems use different sensors to detect subjective uncertainties and their influencing factors in real time, integrate this information with context data and make adjustments that reduce subjective uncertainties. The systemic adaptations of user-focused systems follow a holistic approach that includes the levels of vehicle guidance, interior adaptation and information presentation as well as target guidance are included. By reducing or avoiding subjective uncertainties, the project developments contribute to a positive, comfortable user experience and help to increase the acceptance of ACD. This paper presents research results of AutoAkzept on the topics of user state and activity modelling as well as needs-based adaptation strategies, which represent key components for the implementation of user-focused automation.

## Akzeptanz des automatisierten und vernetzten Fahrens

Die Innovationen automatisierten und vernetzten Fahrens (AVF) führen zu einer Transformation des heutigen Straßenverkehrs. Sie versprechen eine höhere Sicherheit und bezahlbare und bedarfsgerechte Mobilität mit Teilhabe für alle, verbunden mit einem Gewinn an Komfort und nutzbarer Zeit (z. B. [1]). Damit AVF diese Versprechen einlösen kann, müssen nicht nur die technischen Herausforderungen von AVF bewältigt werden, sondern die entwickelten Systeme für AVF auch hohe Verbreitung finden. Dies kann nur gelingen, wenn diese Systeme auf die Akzeptanz ihrer Nutzenden stoßen [2] und zwar im Sinne zukünftiger, aktiver entscheidungsbasierter Nutzung statt nur reaktiver Duldung (siehe [3]).

Die Frage, wann Menschen neue Technologien und technische Systeme akzeptieren bzw. ablehnen, beschäftigt mindestens zwei verschiedene Forschungsrichtungen [4]. Zum einen die Forschung zu Informations- und Kommunikationstechnologien (IKT) und zum anderen die Forschung zu Kognitiver Ergonomie in der Systemgestaltung (KE). Aus dem Bereich IKT-Forschung stammt das prominenteste Modell zur Technikakzeptanz, das Technology Acceptance Model (TAM) von Davies [5] und seine Erweiterungen TAM2 und TAM3 von Venkatesh et al. [6, 7]. Die TAM-Modelle postulieren zwei zentrale Faktoren für die Herausbildung von Akzeptanz, die wahrgenommene Nützlichkeit (*perceived uselfulness*) und die wahrgenommene Einfachheit der Bedienung (*perceived ease of use*). Beide Faktoren sind Determinanten für die Herausbildung einer Nutzungsintention und beeinflussen die Akzeptanz für ein System in Form tatsächlichen Nutzungsverhaltens. Die wahrgenommene Nützlichkeit und Einfachheit der Bedienung werden wiederum durch verschiedene Eingangsvariablen moduliert, welche auf einer, der direkten Nutzung übergeordneten Ebene externe Faktoren wie soziale, berufliche oder organisatorische Einflüsse und Erwartungen widerspiegeln und damit selbst direkte Bestimmungsgrößen der Akzeptanz darstellen. Im Rahmen der KE-Forschung werden dagegen Faktoren in den Mittelpunkt gestellt, die auf Ebene des gemeinsamen zielgerichteten Zusammenwirkens von Menschen und Technik Einfluss auf die Überzeugungen und Wahrnehmungen der Nutzenden nehmen. Wesentlich für die Herausbildung von Akzeptanz technischer Systeme ist demnach zum einen eine Kompatibilität von Technik, Aufgabenstellungen und Anwendungskontexten. Zum anderen müssen Nutzende, darauf aufbauend, Überzeugungen zu Eigenschaften der Technik und dem gemeinsamen Zusammenwirken entwickeln, die wiederum zur Herausbildung von Vertrauen beitragen und schließlich zur Nutzung führen [8].

Auch wenn beide vorgestellten Ansätze wichtige Faktoren für die Herausbildung von Akzeptanz der Nutzenden technischer Systeme benennen, zeigen sich Schwächen hinsichtlich der skizzierten Herausforderung: Ziefle kritisierte z. B., dass die Modelle zur Technikakzeptanz vornehmlich den Arbeitskontext adressieren und die Komplexität der in die Akzeptanzbewertung einfließenden Größen unterschätzen, weshalb sie nur unzureichend auf andere Anwendungskontexte und Technikformen übertragbar sind [9]. Sie verwies in ihrer Kritik auch auf die Bedeutung der Perspektive von Nutzenden und ihren Rollen sowie damit verbundener Kontrollerwartungen, die zu berücksichtigen sind. Ghazizadeh et al. verwiesen auf Forschungsergebnisse, die im Widerspruch zu den Annahmen der KE-Modelle stehen [4]. Denn obwohl hohe Leistungsfähigkeit technischer Systeme und Akzeptanz von Nutzenden in Übereinstimmung mit den KE-Modellen korrelierten (siehe [10]), zeigte sich, dass Nutzende solche Systeme, die ihre Leistungen verbesserten, in etlichen Fällen zugunsten von Systemen ablehnten, die weniger ausgeprägte Vorteile boten [11–13]. Dies zeigt, dass höhere Automatisierungsgrade trotz verbesserter Performanz nicht gleichermaßen von höherer Akzeptanz begleitet werden [14]. Die sich aus der tatsächlichen Nutzung ergebenden Einflüsse auf das Vertrauen und die Bewertung von Systemen müssen entsprechend Berücksichtigung finden. Huang und Haried schließlich kritisierten, dass die auf dem TAM-Modell basierende IKT-Forschung zu Technikakzeptanz nur eingeschränktes Erklärungsvermögen besitzt und das TAM wesentliche Faktoren der Akzeptanzbildung nicht berücksichtigt [15] (siehe auch [16]). Ihre Kritik richtet sich im Kern gegen ein rein rationales Verständnis von Akzeptanzbildung, dass davon ausgeht, dass Menschen eine Reihe kognitiver Prozesse durchlaufen und dabei logische Entscheidungen treffen, ohne dass Emotionen oder das affektive Erleben bei der Nutzung technischer Systeme in irgendeiner Weise Beachtung finden.

Die Forschung und Entwicklung von AVF sollte diese Erkenntnisse berücksichtigen und zur Gewährleistung von Akzeptanz für die zu entwickelnden Systeme erstens den jeweiligen Anwendungskontext und die resultierende Perspektive der Nutzenden und zweitens die Einflüsse, die mit tatsächlicher Nutzung verbunden sind, d. h. das Erleben der Nutzenden und damit verbundene affektive Prozesse, wie das Erleben von Stress, auf die Akzeptanzbildung beachten. Tatsächlich können diese Aspekte zu einer einheitlichen Perspektive der Förderung von Akzeptanz von AVF-Systemen integriert werden. Diese Perspektive wurde im Projekt AutoAkzept [17, 18] verfolgt, dass sich mit subjektiven Unsicherheiten von Nutzenden von AVF befasste.

## Subjektive Unsicherheit und Vertrauen

AVF verändert die Rolle der Menschen beim Fahren. Hoch- und vollautomatisierte Funktionen von Fahrzeugen sollen zukünftig alle Kontroll- und Überwachungsaufgaben übernehmen, die bisher von Menschen ausgeführt werden. Der daraus resultierende Verlust an Kontrolle kann bei den Nutzenden zukünftiger automatisierter Fahrzeuge jedoch Ungewissheit und subjektive Unsicherheit [19] auslösen und zu einem Verlust an Vertrauen führen [20]. Insbesondere, wenn das Nutzungsversprechen die Möglichkeit zur vollständigen Hinwendung auf fahrfremde Tätigkeiten umfasst, kann sich der versprochene Gewinn von AVF für zukünftige Nutzende aber nur einstellen, wenn die Nutzung entsprechender Fahrzeuge (mit den Automationsstufen SAE 4 oder 5, [21]) nicht mit subjektiver Unsicherheit (Ungewissheit) und fehlendem Vertrauen verbunden ist [22].

Subjektive Unsicherheiten treten immer dann auf, wenn Menschen aufgrund fehlender Informationen keine Vorhersagen über den weiteren Verlauf einer Situation oder zukünftige Ereignisse machen können [23]. Jüngere Arbeiten mit Bezug zu AVF bestätigen dies. Es zeigte sich, dass die Geschwindigkeits- und Manöverwahl von Fahrzeugen bei Personen Verstehens‑, Antizipations- und Bewertungsunsicherheit auslösen können [24–26]. Zudem kann die Beschäftigung mit fahrfremden Tätigkeiten, z. B. die Arbeit im Mobile Office, Unsicherheiten darüber erzeugen, ob die verfügbare Zeit bis zum Erreichen einer Systemgrenze ausreicht, notwendig zu bearbeitende Aufgaben fertigzustellen. Dies kann dazu führen, dass sich das Versprechen zum qualitativen Zeitgewinn durch die (zeitweise) Abgabe von Kontrolle an die Automation nicht einlöst, da durch die vorhandene Unsicherheit hinsichtlich der Verfügbarkeit benötigter Zeit für die unterbrechungsfreie Aufgabenbearbeitung Stress und begleitend negative Emotionen ausgelöst werden.

Das Erleben subjektiver Unsicherheiten und der sie begleitenden Emotionen aber vermindert bzw. behindert die Herausbildung von Akzeptanz seitens der Nutzenden, welche maßgeblich durch Vertrauen und subjektive Sicherheit bestimmt wird, die Nutzende bei der Interaktion mit den automatisierten Fahrzeugen empfinden [27–29]. Entscheidend ist hierbei, dass in die Herausbildung von Vertrauen zwar sowohl rationale Prozesse als auch affektives Erleben involviert sind, vor allem aber das emotionale Erleben bestimmt, ob Nutzende Vertrauen entwickeln [30]. Oder, wie Lee und See es fassen: Nutzende denken nicht nur über Vertrauen nach, sie fühlen es [8]. Der Stress und die negativen Affekte, die das emotionale Erleben von Nutzenden beim Auftreten subjektiver Unsicherheiten begleiten, wirken sich damit direkt negativ auf die Vertrauensbildung aus.

Die direkte Erfahrung mit AVF *muss* deshalb die Ausbildung von Vertrauen unterstützen, indem das Auftreten von subjektiven Unsicherheiten minimiert wird. Dafür müssen bei der Systemgestaltung für AVF grundlegende Bedürfnisse (*needs*) von Menschen berücksichtigt werden. Aktuelle Arbeiten verweisen darauf, wie wichtig z. B. die Berücksichtigung von Informationsbedürfnissen (*information needs*) von Nutzenden und verkehrlichen Interaktionspartnern automatisierter Fahrzeuge ist [30–33]. Im Projekt AutoAkzept wurden deshalb die Bedürfnisse von AVF-Nutzenden in den Mittelpunkt gestellt und Lösungen zur Reduktion subjektiver Unsicherheiten auf Basis nutzerfokussierter Systeme entwickelt.

## Herausforderungen für die Systemgestaltung

Werden beim Entwurf und der Gestaltung automatisierter Systeme grundlegende Bedürfnisse von Menschen nicht berücksichtigt, besteht die Gefahr, dass die entwickelten Systeme intransparent erscheinen oder es tatsächlich sind.

**Figure 1:**
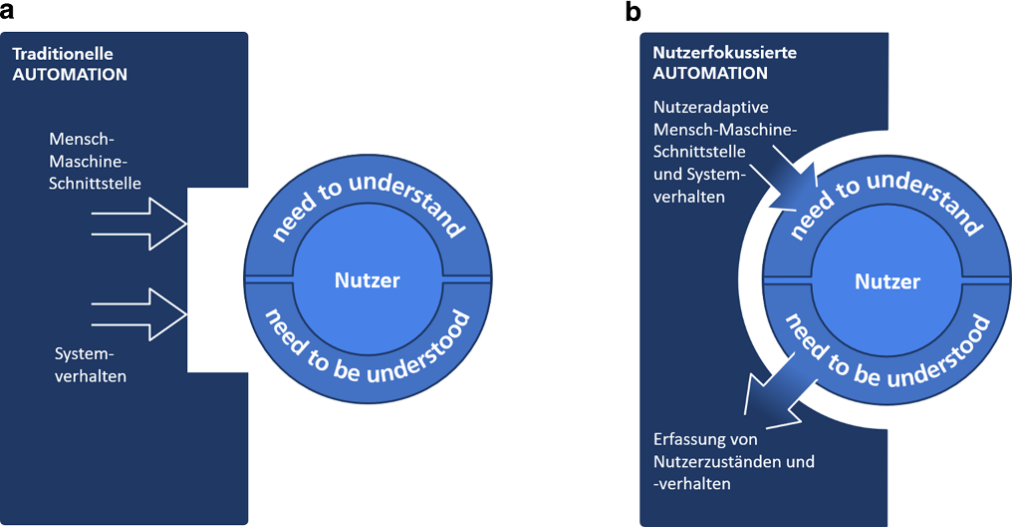

Die Folge ist, dass Personen, die mit solchen Systemen interagieren, die Gründe für das Verhalten einer Automation nicht nachvollziehen oder zukünftige Aktionen nicht vorhersagen können. Die Effekte eines solchen nachteiligen Gestaltungsansatzes werden häufig zusätzlich dadurch verstärkt, dass sich die Nutzenden in der Interaktion mit dem technischen System an die maschinelle Kommunikationsweise anpassen müssen. Diesen nachteiligen Aspekt von Technik- und Systemgestaltung hat beispielsweise die Ethik-Kommission „Automatisiertes und Vernetztes Fahren“ kritisiert [34]. Derart gestaltete Systeme bergen das Risiko, dass Menschen bei der Techniknutzung subjektive Unsicherheiten erleben, verbunden mit den negativen Konsequenzen für ihre Akzeptanz und Nutzungsintention. In Abb. 1a ist die aus diesem Gestaltungsansatz resultierende Systemgestaltung, hier bezeichnet als traditionelle Automation, dargestellt. Schematisch verdeutlicht wird die mit diesem Ansatz verbundene geringe Passung zwischen Menschen (Nutzende) und Maschine (Automation) durch die den Nutzenden nur unzureichend berücksichtigende Kommunikations- bzw. Interaktionsgestaltung über die Mensch-Maschine-Schnittstelle bzw. das Systemverhalten. Um den damit verbundenen negativen Konsequenzen vorzubeugen, wurde im Rahmen von AutoAkzept der Ansatz einer nutzerfokussierten Automation entwickelt. In Abb. 1b ist dieser Ansatz dem traditionellem Gestaltungsansatz gegenübergestellt. Schematisch wird hier die Zielrichtung einer größeren Passung zwischen Menschen (Nutzende) einerseits und der Gestaltung automatisierter Systeme (Automation) andererseits verdeutlicht. Der Ansatz nutzerfokussierter Automation stellt zwei grundlegende menschliche Bedürfnisse in den Mittelpunkt der Systemgestaltung: das Bedürfnis, zu verstehen (*need to understand*, [35]) und das Bedürfnis, verstanden zu werden (*need to be understood*, siehe [36, 37]). Das Bedürfnis, zu verstehen (BZV), ist von zentraler Bedeutung für die erfolgreiche, zielgerichtete Interaktion mit der Umgebung oder einem technischen System. Es ist eng verbunden mit den *information needs* (s. oben) und bildet die Grundlage für den Erwerb und die Anwendung von Wissen, welches den Objekten und Phänomenen der Welt Bedeutung verleiht. Es ermöglicht ein Verständnis über Dinge, Zusammenhänge und Wirkungsweisen und erlaubt die Vorhersage von Ereignissen. Um dem BZV Rechnung zu tragen, muss die Gestaltung automatisierter Systeme sicherstellen, dass die Technologien und technischen Funktionen nicht nur tun, was sie zu tun versprechen, sondern auch, was die Nutzenden sich vorstellen. Automatisierte Systeme, die akzeptiert und bereitwillig genutzt werden sollen, müssen sich deshalb vorhersagbar verhalten, auch dann, wenn sie zuvor noch nie genutzt wurden. Die Umsetzung dieser Anforderung gewährleistet, dass automatisierte Systeme für ihre Nutzenden transparent sind, und zwar so, dass sie Funktionen und Funktionsweisen leicht ableiten können und mit geringstem Aufwand verstehen, wie ein System funktioniert.

Das Bedürfnis, verstanden zu werden (BVZW), ist hingegen unerlässlich, um eine Beziehung aufzubauen. Es muss Berücksichtigung finden, damit Menschen sich gut, gesehen und respektiert zu fühlen. Die Beachtung des BVZW ist eine wichtige Grundlage für das Entstehen von Sympathie und Vertrauen, sowie für das Erleben positiver Affekte und die Abmilderung negativer Einflüsse. Für die Gestaltung von automatisierten Systemen, wie hoch- und vollautomatische Fahrzeuge, hat dies zur Konsequenz, dass sie z. B. erkennen sollten, ob ihre Nutzenden unsicher oder gestresst sind und entsprechend darauf reagieren. Voraussetzung für solche spezifischen, auf den individuellen Nutzenden gerichteten Reaktionen ist jedoch, dass die Systeme erkennen, wann es angebracht ist, z. B. zusätzliche Informationen bereitzustellen und wann nicht. Zu diesem Zweck ist es wichtig, die Nutzenden zu fokussieren und in der Lage zu sein, die unterschiedliche Natur verschiedener menschlicher Zustände sowie daraus folgende Bedürfnisse zu berücksichtigen. In Abb. 1b sind diese Aspekte durch einen Wirkkreis aus Erfassung von Zuständen der Nutzenden, und Anpassung der Mensch-Maschine-Interaktion bzw. des Systemverhaltens an die Nutzenden dargestellt. Die Erfassung der Zustände von Nutzenden ist hierbei die Voraussetzung für die Berücksichtigung des BVZW. Die Möglichkeit zur Anpassung der Mensch-Maschine-Interaktion ist die Voraussetzung dafür, dem BZV gerecht zu werden. Seine Wirksamkeit entfaltet der Gestaltungsansatz, wie in Abb. 1 dargestellt, also nur durch das Zusammenspiel beider Bausteine. Zusammengefasst gilt, dass nutzerfokussierte Systeme sich dadurch auszeichnen, dass sie die Nutzenden in ihrem Verhalten erfassen und durch Anpassungen reagieren und somit auf die individuellen Bedürfnisse von Nutzenden eingehen. Dadurch wird das Vertrauen von Nutzenden in die technischen, insbesondere die hoch- und vollautomatisierten Systeme (SAE 4 und 5) gestärkt. Ihre Akzeptanz steigt.

Der Entwurf nutzerfokussierter Systeme unterscheidet sich von anderen theoretischen Ansätzen durch die explizite Betonung, Beschreibung und Fokussierung zu berücksichtigender Bedürfnisse von Nutzenden. Während die Informationsbedürfnisse der Nutzenden von AVF zwar bereits in der Literatur diskutiert werden (s. Beispiele oben), finden sich bisher keine Arbeiten zum hier beschriebenen BVZW o. ä. In der Forschung zu Akzeptanz und Vertrauen adressieren zu wenige Arbeiten die Erlebensebene. Zahlreiche aktuelle Projekte und damit verbundene Arbeiten im Bereich der Forschung zum automatisierten Fahren widmen sich zwar der Erfassung von Fahrern, insbesondere zur sicheren Gestaltung von Prozessen der Übergabe der Fahrzeugkontrolle z. B. für SAE 3 Anwendungsszenarien [38–40], oder einer komfortablen Gestaltung der Aufgabenverteilung beim Zusammenwirken von Fahrer und Fahrzeug [41]. Die explizite Zielstellung der Verbesserung des affektiven Erlebens von Menschen bei der Nutzung von automatisierten Systemen, zur Gewährleistung von Akzeptanz und Vertrauen, durch Berücksichtigung grundlegender Bedürfnisse auf individueller Ebene, wie dem BVZW, wird dagegen in der Forschung bisher kaum adressiert. Die aktuelle Diskussion zu den Herausforderungen der Gestaltung der Interaktion von Menschen und intelligenten Systemen zeigt jedoch, die Notwendigkeit solche Systeme auch im Sinne menschlicher Wertvorstellungen zu gestalten [42]. Dazu zählt, wesentliche Bedürfnisse von Menschen in ihren individuellen, aktuellen Ausprägungen zu erfassen und in der Interaktion zu berücksichtigen.

## Lösungsbausteine

Im Folgenden werden zwei prototypische Lösungsbausteine vorgestellt, die im Projekt AutoAkzept entwickelt wurden und einen Beitrag zur Entwicklung nutzerfokussierter automatisierter Systeme darstellen. Durch die Covid-19-Pandemie und die damit verbundenen Sicherheitsmaßnahmen kam es zu Einschränkungen für die zwingend auf Studien mit Teilnehmenden angewiesene Projektarbeit in AutoAkzept. Dadurch konnten nicht alle Arbeiten zur Projektlaufzeit finalisiert und integriert werden. Es werden daher zwei unabhängige Arbeiten zu zwei verschiedenen Anwendungsfällen hochautomatisierten Fahrens (SAE 4) vorgestellt. Die Erarbeitung der vorgestellten Lösungsbausteine erfolgte somit jeweils im Rahmen nur eines ausgewählten Anwendungsfalls. Die in den folgenden Abschnitten dargestellten Ergebnisse dienen deshalb in erster Linie dazu, Wege für die Entwicklung von Lösungsbausteinen zu skizzieren, die die Bedürfnisse von Nutzenden automatisierter Systeme, das Bedürfnis, verstanden zu werden (BVZW) und das Bedürfnis, zu verstehen (BZW), berücksichtigen. Die Ergebnisse zeigen exemplarisch, wie die Systemgestaltung nutzerfokussierter Systeme zur Sicherung der Akzeptanz neuer Fahrzeugsysteme im Anwendungsbereich automatisierten und vernetzten Fahrens mit der Automatisierungsstufe SAE 4 beitragen kann. Gemeinsam ist den Arbeiten, dass sie sich mit dem im Projekt AutoAkzept adressierten Nutzerzustand der subjektiven Unsicherheit befassen. Zuerst wird dazu eine Simulatorstudie (Studie 1) vorgestellt, die das BVZW in den Mittelpunkt stellt. Die Studie liefert Ergebnisse für den ersten Lösungsbaustein, die prototypische Umsetzung einer kontextsensitiven Zustandserfassung von Nutzenden am Beispiel des Anwendungsfalls *Arbeiten im Mobile Office.* Anschließend wird eine Online-Studie (Studie 2) vorgestellt, die sich dem BZV widmet. Sie liefert Ergebnisse zur Gestaltung einer prototypisch umgesetzten Mensch-Maschine-Interaktion zur Unsicherheitsreduktion für den Anwendungsfall *Fahrt im Shared Space*.

Obwohl beide Lösungsbausteine der Arbeit zu verschiedenen Anwendungsfällen im Projekt entstammen, werden sie hier gemeinsam präsentiert, um Möglichkeiten für die technische und gestalterische Umsetzung nutzerfokussierter Systeme zu demonstrieren. Das Konzept nutzerfokussierter Systeme bildet hierbei das verbindende Element, dass die erfolgten Forschungs- und Entwicklungsarbeiten in einen gemeinsamen Rahmen stellt.

### Kontextsensitive Zustandserfassung von Nutzenden

**Figure 2:**
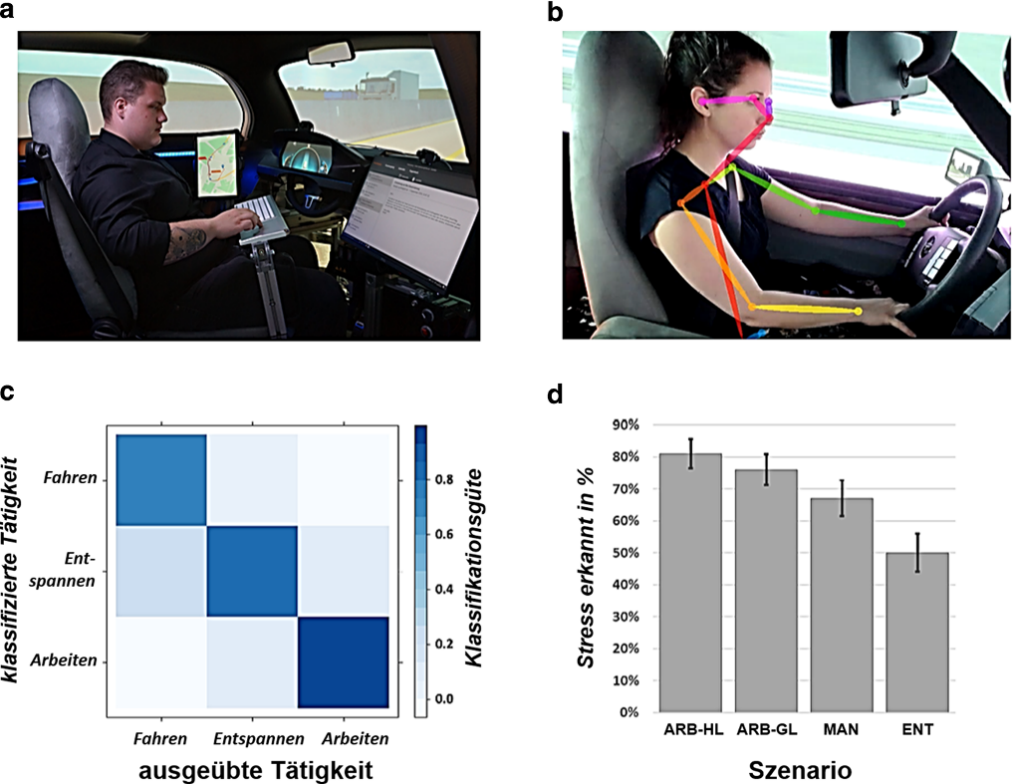

Die kontextbasierte Erfassung der Zustände von Nutzenden wurde in einem Demonstrator im Fahrsimulator Virtual Reality Lab [43] erforscht. Das auf dem Urban Modular Vehicle [44] basierende MockUp, welches zum Einsatz kam, repräsentiert ein automatisiertes Fahrzeug (AF) mit der Automationsstufe SAE 4 [21]. Im Projekt wurde ein Setup erarbeitet, dass die Möglichkeit zum mobilen Arbeiten bietet (siehe [45], Abb. 2a). Es stützt sich auf ein ein‑/ausfahrbares Lenkrad, einen zur Seite drehbaren Fahrersitz, eine klappbare Tastatur sowie einen festinstallierten Monitor. Den Nutzenden des Fahrzeugs bietet sich dadurch die Möglichkeit, die Zeit während einer vollautomatisierten Fahrt z. B. für die computergestützte Bearbeitung anspruchsvoller Aufgaben zu nutzen. Zur Umsetzung des eingeführten Konzeptes einer nutzerfokussierten Automation (siehe Abb. 1) verfügt das Fahrzeuginterieur über Kameras zur Aufnahme des Oberkörpers sowie über in den Sitz integrierte kapazitive Elektroden zur Aufzeichnung von Herzrate und Herzratenvariabilität über ein Elektrokardiogramm [46]. Gestützt auf diese sensortechnische Ausstattung können mit dem Demonstrator automationsbezogene affektive Zustände von Nutzenden, wie Unsicherheit oder Stress und deren kontextsensitive Erfassung, untersucht werden.

Im Anwendungsfall *Arbeiten im Mobile Office*, welcher mit dem Demonstrator adressiert wurde, verfügt das AF über einen Autobahnpilot und kann somit Autobahnfahrten automatisiert, ohne notwendige Eingriffe der Fahrer bewältigen. Nach Abfahrt von der Autobahn müssen die Nutzenden die Kontrolle übernehmen und manuell im urbanen oder ländlichen Raum fahren. Durch diese Situation der notwendigen Kontrollübergabe an der Systemgrenze kann für Nutzende, welche zuvor Arbeitstätigkeiten, etwa das Fertigstellen einer wichtigen Kundenpräsentation oder die Beantwortung dringender Emails, durchführen, subjektive Unsicherheit darüber entstehen, ob die schlecht zu unterbrechende Aufgabe noch rechtzeitig fertiggestellt werden kann. Diese Unsicherheit übt einen Zeitdruck aus, der durch den Nutzenden als Stressor empfunden werden kann und somit Stress auslöst [47]. Stress wird hier definiert als eine Situation, in der ein Individuum die Anforderungen durch die Umwelt so bewertet, dass sie seine eigenen Anpassungsfähigkeiten übersteigen [48]. Da Stress zu einer verstärkten Aktivierung des sympathischen Nervensystems und damit zu einer Erhöhung des physiologischen Erregungslevel führt, eignen sich kardiovaskuläre Indikatoren wie Herzrate und Herzratenvariabilität zur Erfassung von Stress (z. B. [49, 50]). Diese Maße haben darüber hinaus den Vorteil, dass ihre Erfassung durch im Sitz verbaute Sensoren möglich ist, ohne dabei den Nutzenden bei der Bearbeitung von Aufgaben zu stören oder zu beeinträchtigen [46]. Ein nutzerfokussiertes System sollte entstehenden Stress von Nutzenden erkennen und Informationen bereitstellen oder Anpassungen anbieten, die das BVZW der Nutzenden befriedigen. Die Automation könnte, wenn die Randbedingungen es zulassen, z. B. eine Routenanpassung anbieten, die die Kontrollübergabe zeitlich nach hinten verschiebt und gleichzeitig durch lichttechnische Anpassungen im Interieur das fokussierte Arbeiten unterstützen. Um solch ein nutzerfokussiertes Systemverhalten zu ermöglichen, wird eine kontextsensitive Erfassung des Zustands von Nutzenden benötigt. Folglich diente diese Studie der Entwicklung von echtzeitfähigen Methoden zur Erfassung von Stress und der Aktivität von Nutzenden von AFs. Die Aktivität bildet hierbei den kontextuellen Rahmen, in dem der Stress erlebt wird. Die Integration beider Erfassungsmethoden zu einer kontextsensitiven Zustandserfassung bildet eine Grundlage für die Entwicklung nutzerfokussierte Automation und richtet sich auf das BVZW von Nutzenden.

#### Studie 1: Arbeiten im Mobile Office

Für die Entwicklung einer auf maschinelle Lernverfahren gestützten Methode zur kontextsensitiven Erfassung des aktuellen Stresslevels von Nutzenden von AFs für den dargestellten Anwendungsfall (siehe [51]) wurde im Projekt AutoAkzept eine Simulatorstudie durchgeführt. Der dabei erhobene Datensatz umfasst die Daten von insgesamt 32 Versuchsteilnehmenden (18 Frauen, 14 Männer). Das mittlere Alter der Stichprobe lag bei 25 Jahren (*SD* = 7,6), wobei der/die jüngste Teilnehmende 18 und der/die älteste Teilnehmende 62 Jahre alt war. Die Versuchsteilnehmenden erlebten im Demonstrator verschiedene Szenarien: Das erste Szenario beinhaltete eine manuelle Fahrt (MAN) auf einer Autobahn. In drei weiteren Szenarien fuhr das Fahrzeug automatisiert und die Versuchsteilnehmenden mussten entweder eine Bürotätigkeit (Abarbeiten von E‑Mails im E‑Mail-Client, Abb. 2a) mit zwei verschiedenen Arbeitslastniveaus (geringe Last, ARB-GL, oder hohe Last, ARB-HL) erledigen oder konnten sich entspannen (ENT). Jede Fahrt dauerte etwa 15 min. In den automatisierten Fahrten erhielten die Versuchsteilnehmenden die Instruktion, dass sie nach Ablauf dieser Zeit wieder die Kontrolle übernehmen müssen und folglich bis dahin alle E‑Mails im Postfach bearbeitet sein sollten. Somit sollte durch die variierende Aufgabenlast, kombiniert mit der drohenden Übernahme, Zeitdruck und damit Stress bei den Versuchsteilnehmenden induziert werden. ENT sollte dabei keinen Stress erzeugen. Die Aufgaben ARB-GL und ARB-HL waren so gestaltet, dass sie bei den Versuchsteilnehmenden verschiedene Stresslevel induzieren sollten. Während ARB-GL erforderte das Beantworten aller E‑Mails zwar ein kontinuierliches Bearbeiten der Aufgabe; die Häufigkeit der Mails war aber so gewählt, dass die Aufgabenanforderung die Fähigkeiten der Versuchsteilnehmenden nicht übersteigen sollte. ARB-HL wurde hingegen so ausgelegt, dass die vorgegebene Zeit grundsätzlich nicht ausreichte um alle E‑Mails zu bearbeiten, so dass hier eine Situation geschaffen wurde, die bei allen Versuchsteilnehmenden Stresserleben auslösen sollte^1^. Während der Fahrten wurde ein EKG abgeleitet – hier kam für die Studie noch ein mobiles System der Marke HealthLab zum Einsatz – sowie Oberkörper und Kopf der Versuchsteilnehmenden per Kamera von der rechten A‑Säule aus aufgezeichnet. Aus dem EKG wurde zu jedem Herzschlag die Zeit zum vorherigen Herzschlag (Interbeatintervall, IBI) als Indikator für die aktuelle Herzrate der Versuchsteilnehmenden berechnet. Um Informationen über die Körperhaltung zu erlangen, erfolgte eine Extraktion der Positionen relevanter Körperpunkte aus den Videodaten mittels OpenPose [52] in Echtzeit (siehe Abb. 2b).

#### Ergebnisse

Das IBI und die Lokationen der Körperpunkte dienten als Input zur Entwicklung der kontextsensitiven Erfassung des Stresslevels der Nutzenden auf Basis maschineller Lernverfahren. Hierfür wurden aus den IBI-Daten jeweils für überlappende Zeitfenster von 30 s (29 s Überlappung) die mittlere Herzrate sowie die RMSSD („root mean squared difference of sukzessive heart beats“, siehe [53]) als Indikator für die Herzratenvariabilität berechnet. Anschließend erfolgte datengetrieben mittels eines Clusterings über Gaußsche Mischmodelle eine Abschätzung des aktuellen Erregungslevels der jeweiligen Versuchsteilnehmenden. Parallel dazu erfolgte auf Grundlage ausgewählter Körperpunktpositionen der Hände und des Kopfes eine Bestimmung der der aktuellen Haltung (Körperpose) und darauf aufbauend die Klassifikation der Tätigkeit über einen Support Vector Classifier (SVC). Dabei wurde die Annahme zu Grunde gelegt, dass die Szenarien MAN, ENT und ARB-HL jeweils vollständig die Tätigkeiten* Fahren, Entspannen* und *Arbeiten* enthalten. Für das Szenario ARB-GL wurde angenommen, dass es sowohl die Tätigkeit *Arbeiten* als auch *Entspannen* enthält, in Abhängigkeit davon, wie die Versuchsteilnehmenden sich die Aufgabenbearbeitung einteilten. Deshalb erfolgte das Training des SVC mit den Daten der ersten drei Szenarien, so dass jedes Szenario selbst jeweils eine Tätigkeit repräsentierte und ARB-GL nicht für das Training des SVC genutzt wurde. Die Klassifikationsgüte des SVC für die drei Tätigkeiten ist in der Konfusionsmatrix in Abb. 2c dargestellt. Die Ergebnisse zeigen, dass die Erkennung der ausgewählten Aktivitäten über den prototypisch implementierten Ansatz insgesamt sehr vielversprechend ist, mit einer Klassifikationsgüte (Anteil korrekten Klassifikationen einer Tätigkeit) für *Fahren* von 0,75, für *Entspannen* von 0,84 und für *Arbeiten* von 0,93. Herausforderungen für die Erkennung entstehen hierbei z. B. dadurch, dass viele Versuchsteilnehmende in den Szenarien MAN und ENT für die ausgewählten Körperpunkte vergleichbare Positionen wählten (vgl. [51]). Eine Hinzunahme weiter Körperpunkte und Änderungen im Fahrzeugsetup wie flexibel anpassbare Sitzeinstellungen (z. B. für *Entspannen*) können hier zu einer besseren Klassifikationsleistung beitragen.

Basierend auf den Werten für Erregung und Haltung wurde, jeweils in Fenstern von 60 s, bestimmt, wie hoch die Wahrscheinlichkeit ist, dass die Nutzenden gestresst sind (siehe Abb. 2d) bzw. welche Aktivität sie gerade durchführen. Dies erfolgte durch eine Bestimmung der relativen Häufigkeit der bestimmten Erregung bzw. der einzelnen Posen in dem jeweiligen Zeitfenster. Die Wahrscheinlichkeit für Stress errechnet sich folglich daraus, wie oft der Klassifikator in der letzten Minute ein hohes Erregungslevel erkannt hat. Im aktuellen Ansatz wurde der Grenzwert so gewählt, dass Stress angenommen wird, wenn in mehr als 50 % der vergangenen Minute ein hohes Erregungsniveau vorhanden ist. Mit dem gewählten Ansatz wurde über alle Versuchsteilnehmenden erwartungskonform am häufigsten im Szenario ARB-HL Stress erkannt (81 % der Zeit), gefolgt von ARB-GL (76 %), MAN (67 %) und ENT (50 %). Eine Erklärung für die relativ hohen Werte für ENT und MAN bietet das experimentellen Setting, bei dem die Versuchsteilnehmenden unter Beobachtung stehen und in einem kurzen Zeitraum einer Vielzahl unbekannte neuer Reize und Eindrücke ausgesetzt sind.

Hierbei ist anzumerken, dass Stress eigentlich als Kontinuum aufgefasst wird und somit die hier verwendete Diskretisierung (in Stress vorhanden oder nicht) in Frage gestellt werden kann. Letztlich würde das hier gewählte Modell zur Bestimmung von Stress auf Basis des subjektiven Erregungslevels auch erlauben einen kontinuierlichen Stresswert auszugeben. Es sollte daher jeweils für den konkreten Anwendungsfall geschaut werden, welche Ausgabe dem Anwendungszweck dient. Eine vollständige Darstellung der Details zum Aufbau und der Leistungsfähigkeit des vorgestellten Ansatzes bietet [51].

Durch die Abschätzungen für das aktuelle Stresslevel sowie die aktuelle Aktivität können nun nicht nur Aussagen darüber getroffen werden, ob die Nutzenden aktuell ein hohes Stresslevel haben, sondern auch, aus welcher Aktivität detektierter Stress wahrscheinlich resultiert. Damit wird es möglich das BVZW der Nutzenden zu berücksichtigen und diesem Bedürfnis durch Anpassungen des Systemverhaltens gezielt gerecht zu werden. Das bedeutet, dass auf Grundlage der kontextsensitiven Zustandserfassung den Nutzenden Interventionsstrategien zur Unterstützung angeboten werden können, die es ermöglichen ihr Erleben gezielt positiv zu beeinflussen bzw. sie gezielt in ihrem Handeln zu unterstützen. Dafür ist es notwendig Informationen zum Zustand von Nutzenden mit Informationen zum Kontext zu integrieren. Denn Nutzende, deren Stresserleben aus der Arbeit im Mobile Office herrührt, haben einen anderen Unterstützungsbedarf als jene, deren Stress beim bzw. durch das Fahren entsteht. D. h. nur solche systemischen Anpassungen, die eine hohe Passung mit dem aktuellen Zustand und Kontext von Nutzenden aufweisen, befriedigen ihre individuellen Bedarfe und damit ihr BVZW. Für die Tätigkeit im Mobile Office etwa, könnte eine Anpassung der Beleuchtung unter Nutzung moderner Lichttechnik helfen, um die Konzentration zu fördern [39] und damit das fokussierte Arbeiten zu erleichtern. Die aktuelle Aktivität der Nutzenden ist jedoch nur ein relevanter Kontextfaktor. Informationen über die Umgebung, die Verkehrssituation oder die aktuell gefahrene Route und mögliche Alternativen können das Verständnis über die Ursache(n) des aktuellen Zustands der Nutzenden von AFs weiter verbessern und so zu ihrer Unterstützung und Verbesserung ihres Erlebens beitragen [18].

### Unsicherheitsreduktion über das internale HMI

Im Rahmen einer Online-Studie wurden in AutoAkzept für den Anwendungsfall *Fahrt im Shared Space* Gestaltungsmaßnahmen zur Reduktion subjektiver Unsicherheiten untersucht. Für die Reduktion der subjektiven Unsicherheit ist es von entscheidender Bedeutung, den Nutzenden eines automatisierten Systems das aktuelle und zukünftige Verhalten der Automation möglichst transparent, effizient und effektiv zu kommunizieren. Hierbei soll eine leicht verständliche und eindeutig gestaltete Mensch-Maschine-Interaktion (MMI) die nötige Unterstützung von Nutzenden eines AFs, mit Hilfe einer informationsvermittelnden, im Fahrzeug integrierten, d. h. internalen Mensch-Maschine-Schnittstelle (*internal human-machine interface*, iHMI), gewährleisten. Durch eine kontinuierliche Erfassung des Zustands und Verhaltens von Nutzenden soll jederzeit eine Anpassung der MMI an die Bedürfnisse der Nutzenden möglich sein. Ziel ist es, durch ein adaptives iHMI die individuellen Informationsbedürfnisse der Nutzenden optimal adressieren zu können und dadurch ihrem BZV gerecht zu werden. Dazu soll bei Auftreten einer subjektiven Unsicherheit von Nutzenden eine sofortige subjektive Sicherheitssteigerung mittels intelligenter Anpassung des MMI über das iHMI ermöglicht werden. Dies ist besonders während der automatisierten Fahrt von Bedeutung, da die Nutzenden von AFs die Fahrzeugführung an die Automation abgeben und die Rolle eines Fahrgastes übernehmen. Dieser Rollenwechsel bedingt ebenfalls eine Anpassung der MMI. Die Nutzenden müssen sich in der neuen Rolle als Fahrgast verstanden fühlen und benötigen nun andere Informationen, damit ihr BZV befriedigt wird und sie sich während der Fahrt sicher fühlen [54]. Adaptive iHMI-Lösungen bilden daher einen weiteren Baustein für die Entwicklung nutzerfokussierter Automation, der in diesem Rahmen auf das BZV der Nutzenden gerichtet ist.

Um die subjektive Unsicherheit der Nutzenden in einem AF möglichst gering zu halten bzw. in komplexen Verkehrssituationen zu reduzieren, wurde in der aktuellen Studie auf ein bereits in einem anderen Nutzungskontext erprobtes iHMI-Konzept [55] zurückgegriffen. Hierbei wird mit Hilfe eines im Fahrzeuginnenraum verbauten 360°-LED-Lichtbandes eine MMI mithilfe von richtungsaufgelösten Lichtsignalen realisiert. Diese MMI-Lösung wurde sowohl für die Fahrerunterstützung während manueller Autobahnfahrten ohne Automationseingriffe (SAE 0), als auch in höheren Automationsstufen evaluiert [56]. Besonders in den höheren Automationsstufen (> SAE 2) soll den Fahrenden mittels intelligenter Systemrückmeldung des iHMI verdeutlicht werden, welche relevanten, d. h. mit dem eigenen Fahrzeug interagierenden, Verkehrsteilnehmenden die Automation wahrgenommen und bei ihrer Handlungsplanung berücksichtigt hat. Hierdurch soll die Transparenz des Automationsverhaltens erhöht und so eine Reduktion der subjektiven Unsicherheit der Fahrenden erreicht werden. Einen Transfer des iHMI-Konzepts für hochautomatisierte Fahrzeuge (SAE 4) im urbanen Verkehrsraum wurde ebenfalls [57] realisiert und sollte in dieser Studie weiter evaluiert werden. Im Mittelpunkt stand hierbei der Einfluss einer höher-komplexen und schwieriger vorhersehbaren Verkehrsumgebung auf mögliche subjektive Unsicherheiten der Nutzenden von AFs.

Die im Folgenden dargestellte Untersuchung soll die Forschungsfrage bezüglich des optimalen Darbietungszeitpunkts bzw. der Darbietungsdistanz eines adaptiven iHMI untersuchen. Die Konkrete Forschungsfrage der Untersuchung lautet daher: Zu welchem Zeitpunkt bzw. bei welcher Distanz wünschen sich die Nutzenden eines hochautomatisierten Fahrzeugs (SAE 4) eine Rückmeldung zu anderen Verkehrsteilnehmenden durch ihr iHMI? Zur Beantwortung dieser Forschungsfrage wurden die Teilnehmenden der Online-Studie eingeladen, als Co-Designende für ein adaptives iHMI ihre Informationsbedürfnisse hinsichtlich dieser Fragestellung direkt in die iHMI-Interaktionsgestaltung einzubringen. Die für nutzerfokussierte Systeme erforderliche Zustandserfassung von Nutzenden wurde damit quasi simuliert und durch die Bewertung der Studienteilnehmer ersetzt. Zusätzlich wurde im Rahmen der Online-Studie auch die User Experience (UX) für die Interaktion der Nutzenden mit diesem im Co-Design-Prozess entstandenen iHMI untersucht.

Insgesamt nahmen 106 Versuchsteilnehmende (38 Frauen, 65 Männer, einmal divers und zweimal ohne Angabe des Geschlechts) an der Online-Studie teil. Das mittlere Alter der Stichprobe lag bei 34,16 Jahren (*SD* = 12,54), wobei der/die jüngste Teilnehmende 18 und der/die älteste Teilnehmende 69 Jahre alt war. Da bei hochautomatisierten Fahrzeugen (SAE 4) der Mensch nicht mehr als Rückfallebene fungieren muss, wenn die Automation an ihre Grenzen gerät, konnten sich auch Erwachsene ohne Führerschein und geringer jährlicher Fahrpraxis an der Studie beteiligen. Bis auf drei Versuchsteilnehmende waren alle Studienteilnehmende im Besitz einer gültigen Fahrerlaubnis und fuhren im Mittel 8949 km (*SD* = 8809) pro Jahr. Alle Versuchsteilnehmende gaben an, bereits von automatisierten Fahrzeugen gehört zu haben. Darüber hinaus interessieren sich 70 % der Versuchsteilnehmenden stark bis sehr stark für das Thema automatisiertes Fahren. Um die Technikaffinität der Stichprobe einschätzen zu können, wurde ein Fragebogen zur interaktionsbezogenen Technikaffinität (ATI) [58] als Messmethode verwendet. Der Fragebogen erfasst die Interaktion mit technischen Systemen auf einer sechsstufigen Skala von 1 = „stimmt gar nicht“ bis 6 = „stimmt völlig“. Die Ergebnisse zeigten eine eher hohe Technikaffinität in der Gesamtstichprobe (*M* = 4,40; *SD* = 0,88). Die Akquirierung der Versuchsteilnehmenden erfolgte über die Bekanntmachung der Online-Studie via LinkedIn, Twitter, Mailing-Listen und direkten Kontakt per Email mit Versuchsteilnehmenden der DLR-Testfahrerdatenbank. Die Teilnahme an der Online-Studie erfolgte auf freiwilliger Basis und ohne Bezahlung. Die Teilnehmenden konnten sich jedoch an einem Gewinnspiel beteiligen, bei dem vier Amazon-Gutscheine im Wert von jeweils 25 € verlost wurden.

#### Studie 2: Fahrt im Shared Space

Die Studie wurde in Form einer experimentellen Online-Studie durchgeführt, bei der die Nutzenden als Co-Designende für das nutzerfokussierte, adaptive HMI direkt in den Gestaltungsprozess der Interaktion der Automation mit den Nutzenden einbezogen wurden. Im Mittelpunkt der Untersuchung stand der Zeitpunkt bzw. die Distanz des eigenen AF zu anderen Verkehrsteilnehmenden, bei der sich die Nutzenden eine Informationsrückmeldung über das Erkennen der Automation dieser Verkehrsteilnehmenden über das iHMI wünschen. Hierfür wurden unterschiedliche, in der Grafikengine Unreal Engine 4 erstellte, Videoausschnitte mit einem strukturierten Interview (Fragebogen) kombiniert. Die Gesamtdauer der Studie lag bei durchschnittlich 20 min (*SD* = 6). Zur Untersuchung der Forschungsfrage wurde ein einfaktorielles Untersuchungsdesign mit Messwiederholung verwendet. Als unabhängige Variable (UV) wurde die Art des verkehrlichen Interaktionspartners in den dargebotenen Videos in einer dreifachen Stufung (männlicher erwachsener Fußgänger vs. männlicher erwachsener Fahrradfahrer vs. weibliches Kind) systematisch manipuliert. Alle Versuchsteilnehmenden erlebten und beurteilten sämtliche Ausprägungen der UV. Als abhängige Variable (AV) wurde zum einen die individuelle Distanz erhoben, bei welcher sich die Versuchsteilnehmenden eine Systemrückmeldung wünschen (BZV), um keine Unsicherheit in der Interaktion des hochautomatisierten Fahrzeugs (SAE 4) mit einem anderen Verkehrsteilnehmenden zu verspüren. Zum anderen wurde der bevorzuge Zeitpunkt für eine Systemrückmeldung des iHMI mittels eines Items näher bestimmt, in Bezug auf Sichtbarkeit von anderen Verkehrsteilnehmenden sowie in Bezug auf den Bremsvorgang des eigenen hochautomatisierten Fahrzeugs. Um die ganzheitliche User Experience (UX) des Anzeige- und Interaktionsdesigns zu bestimmen, wurde der standardisierte Fragebogen UEQ‑S [59] in die Untersuchung integriert. Hierbei konnten die Versuchsteilnehmenden auf einer siebenstufigen Skala mit semantischen Differenzialen ihre Einschätzung zur UX des HMI auf den Subskalen der pragmatischen und hedonischen Qualität bewerten. Diese Bewertung bezog sich auf das iHMI inklusive der individuell von den Teilnehmenden als Co-Designer eingestellten Systemrückmeldungen über erkannte andere Verkehrsteilnehmende, also ganz im Sinne eines nutzerfokussierten HMI.

**Figure 3:**
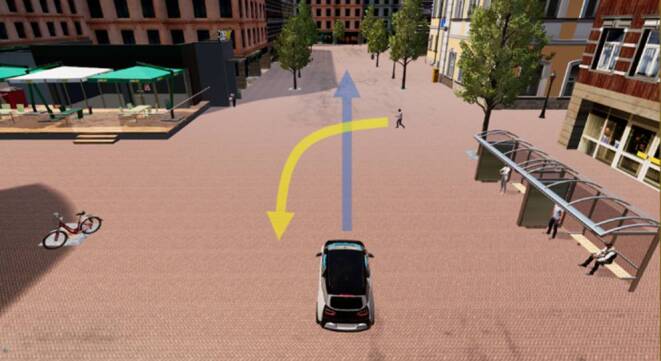

Die Abfolge der verkehrlichen Interaktionspartner wurde über alle Versuchsteilnehmende konstant gehalten. Der erwachsene Fußgänger stellte den ersten Interaktionspartner dar. Darauf folgte der erwachsene Fahrradfahrer und abschließend die Interaktion mit einem Kind. Da von einem stärkeren Grad der Fahrgastverunsicherung im Kontakt mit schwächeren Verkehrsteilnehmenden ausgegangen werden kann, wurden für die hier vorliegende Studie ausschließlich schwache bzw. verletzliche Verkehrsteilnehmende verwendet. Darüber hinaus wurde durch eine Interaktion mit einem Kind ein besonders schützenswerter und in seinen Handlungen eher unvorhersehbarer Verkehrsteilnehmer in die Studie inkludiert. Die Autoren gehen von einem starken Einfluss des besonders schützenswerten Verkehrsteilnehmers (Kind) auf die empfundene subjektive Unsicherheit und den daraus resultierenden Informationsbedarf des AF-Nutzenden aus. Dieser starke Einfluss des Kindes als Interaktionspartner im Verkehr wäre möglicherweise ein unerwünschter extremer Anker gewesen (auch bei Randomisierung), der alle folgenden Bewertungen hätte beeinflussen können. Um diesen möglichen Ankereffekt auszuschließen, wurde eine starre Reihenfolge der Verkehrsteilnehmer gewählt, die in der Interaktion mit dem Kind kulminierte. Als Verkehrsumgebung wurde ein Shared Space ausgewählt (Abb. 3). Hierbei handelt es sich um einen wenig regulierten Verkehrsraum, der keine klaren Fahrbahnbegrenzungen aufweist und auf dem sich alle Verkehrsteilnehmenden gleichberechtigt bewegen. Lediglich die erlaubte Höchstgeschwindigkeit und die Vorfahrt (rechts vor links) sind auf einem Shared Space reguliert. Die verwendeten Videos versetzten die Versuchsteilnehmende in die Perspektive eines Nutzenden eines hochautomatisierten Fahrzeugs (SAE 4). Für die Darstellung des iHMI wurde ein computergeneriertes Video mit Hilfe der Grafikengine Unreal Engine 4 aus der Perspektive des Innenraums eines hochautomatisierten Fahrzeugs verwendet.

**Figure 4:**
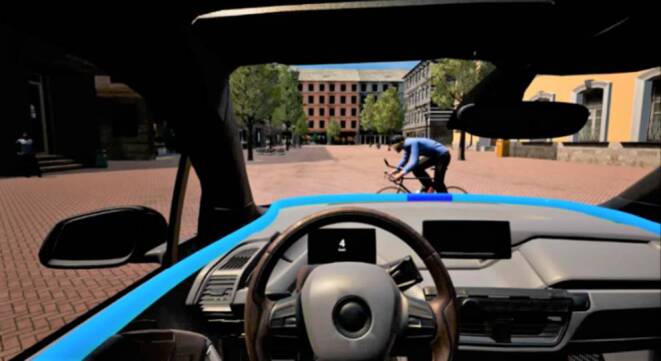

Als iHMI wurde ein im Fahrzeuginnenraum verbautes 360°-LED-Lichtband genutzt. Durch ein blaues Leuchten des 360°-LED-Lichtbands wurde den Nutzenden mitgeteilt, dass sich die Automation im hochautomatisierten Modus (SAE 4) befand und kein eigenes Eingreifen notwendig war (Abb. 4). Zusätzlich kommunizierte das LED-Lichtband als iHMI richtungsaufgelöste Informationen in Form von Lichtsignalen direkt auf dem LED-Lichtband selbst. Hierdurch sollte dem Nutzenden mittels intelligenter Systemrückmeldung des iHMI verdeutlicht werden, welche Verkehrsteilnehmende die Automation wahrgenommen hatte, um das Bedürfnis der Nutzenden, die Automation zu verstehen, zu befriedigen. Für diese Systemrückmeldung des iHMI, wurde ein dunkelblauer Balken auf dem LED-Lichtband angezeigt (Abb. 4). Hierbei wurde der dunkelblaue Balken jeweils unterhalb (aus Perspektive der Nutzenden) des detektierten Verkehrsteilnehmenden angezeigt. Der dunkelblaue Balken folgte dem Verkehrsteilnehmenden positionsgetreu auf dem 360°-LED-Lichtband, um dem Nutzenden kontinuierlich eine Systemrückmeldung über die Position des detektierten Verkehrsteilnehmenden zu geben. Durch diese intelligente Interaktionsstrategie sollte dem Nutzenden verdeutlicht werden, dass detektierte Verkehrsteilnehmende bei der Auswahl zukünftiger Handlungen des AF berücksichtigt werden. Ziel des iHMI war es, die Transparenz des Automationsverhaltens zu erhöhen und so subjektive Unsicherheit der AF-Nutzenden zu reduzieren bzw. zu vermeiden.

Zu Studienbeginn gaben alle Versuchsteilnehmende zunächst ihr Einverständnis zur Aufzeichnung und Verarbeitung ihrer Angaben im Zuge der wissenschaftlichen Forschung. Es folgte ein kurzes Beispielvideo, um die technischen Voraussetzungen des Abspielgeräts der Studienteilnehmenden zu testen. Hiernach folgte die Erfassung demographischer Daten und die Instruktion für den Übungsdurchgang. Vor dem Übungsdurchgang wurden die Versuchsteilnehmenden bezüglich der Funktionsweise des AF und des iHMI aufgeklärt und instruiert, lediglich das Videomaterial zu betrachten. In dem Video des Übungsdurchgangs erlebten die Versuchsteilnehmenden aus der Perspektive des Nutzenden eines AF die Interaktionen zwischen dem AF und einem bevorrechtigen Verkehrsteilnehmenden in einer Rechts-vor-links-Situation auf einem Shared Space. Hierbei durchfuhr das AF mit einer Geschwindigkeit von 20 km/h den Shared Space und begegnete einem kreuzenden Fußgänger. Da der andere Verkehrsteilnehmende aus einer Einmündung auf den Shared Space kam, war er zu Beginn des Videos verdeckt und wurde erst bei einer Distanz von 40 m (AF zu anderen Verkehrsteilnehmenden) sichtbar. Der andere Verkehrsteilnehmende bewegte sich mit einer konstanten Geschwindigkeit von 4,5 km/h auf einer kreuzenden Trajektorie zum AF (Abb. 3). Der von rechts kommende Verkehrsteilnehmende war vorfahrtsberechtigt und das AF musste ihm regelkonform Vorfahrt gewähren. Das AF verringerte hierzu seine Geschwindigkeit und kam schließlich zum Stillstand, um den anderen Verkehrsteilnehmenden queren zu lassen. Sobald der Fahrweg wieder frei war, beschleunigte das AF und setzte seine Fahrt über den Shared Space fort. Kurz darauf endete das Video. Nachdem die Versuchsteilnehmenden in einem ausführlichen Übungsdurchgang das Funktionskonzept des iHMI mit einem fixen Informationsdarbietungszeitpunkt (Beginn der Bremsung des AF, Distanz zum anderen Verkehrsteilnehmer 5 m) betrachten konnten, folgten die Experimentaldurchgänge, in denen die Versuchsteilnehmenden als Co-Designende den für sie optimalen Zeitpunkt der Informationsdarbietung auf dem iHMI definieren konnten. Um dies zu erreichen, wurden alle Versuchsteilnehmende bezüglich ihrer neuen Aufgabe und des Aufbaus der Eingabemaske instruiert. In den Experimentaldurchgängen wurden Videos mit unterschiedlichen Verkehrsteilnehmenden (erwachsener Fußgänger, Fahrradfahrer, Kind), jedoch mit jeweils identischen Geschwindigkeiten der Verkehrsteilnehmenden und einem identischen Ablauf verwendet. Hierbei folgten die Experimentaldurchgänge dem Ablauf des Übungsdurchgangs. Während der Experimentaldurchgänge wurde jedoch kein fixer Informationsdarbietungszeitpunkt auf dem iHMI präsentiert. Die Aufgabe der Versuchsteilnehmenden bestand vielmehr darin, die Videos zu dem für sie individuell optimalen Zeitpunkt einer Systemrückmeldung zu stoppen und die jeweilige Distanz zum anderen Verkehrsteilnehmenden (jeweils im Videomaterial eingeblendet) in ein Antwortfeld einzutragen. Dieser selbsteingestellte Zeitpunkt sollte ihrem Bedürfnis, das Verhalten der Automation zu verstehen und damit ihre subjektive Unsicherheit zu reduzieren, nahekommen. Dieser Vorgang wiederholte sich für jeden der drei Verkehrsteilnehmenden (Erwachsender Fußgänger, Fahrradfahrer, Kind). Zum Abschluss der Untersuchung beantworteten die Versuchsteilnehmenden ergänzende Fragen zum optimalen Zeitpunkt der Systemrückmeldung und beurteilten das im Sinne eines nutzerfokussierten Co-Designs mitgestaltete iHMI bezüglich der wahrgenommenen User Experience (UEQ‑S [59]).

#### Ergebnisse

**Figure 5:**
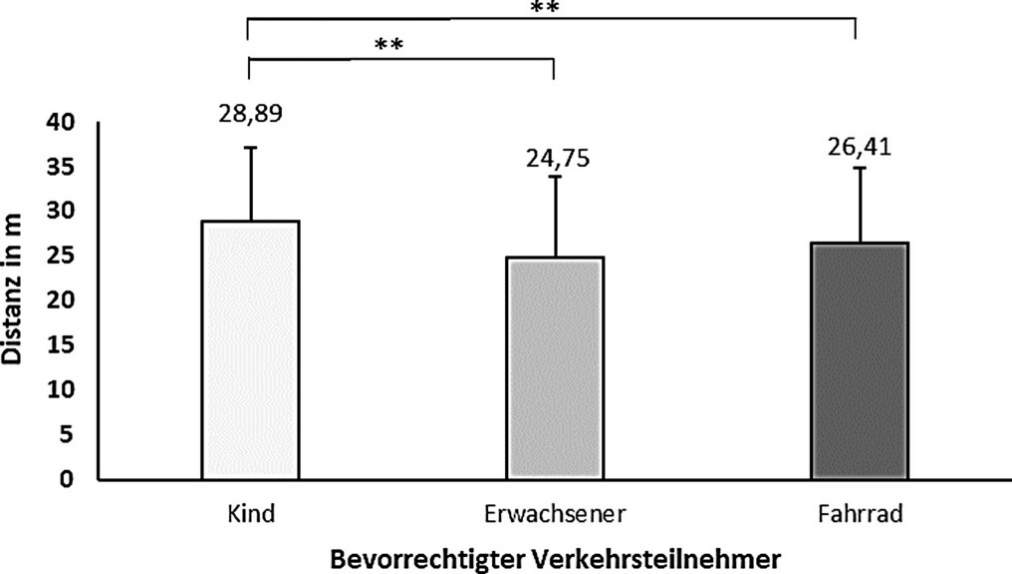

Die erste Fragestellung bezog sich auf den Zeitpunkt bzw. die Distanz, ab welcher sich Nutzende eines AF eine Systemrückmeldung über erkannte, interagierende Verkehrsteilnehmende in ihrer Fahrzeugumgebung wünschten. Durch die Rückmeldung der erkannten Verkehrsteilnehmenden soll die Unsicherheit der Nutzenden bezüglich des zukünftigen Fahrzeugverhaltens der Automation verringert werden. Hierbei konnten die Versuchsteilnehmenden den individuellen Zeitpunkt der Rückmeldung frei wählen und auf ihre Bedürfnisse anpassen. Da auch die Art der interagierenden Verkehrsteilnehmenden einen Einfluss auf das individuelle Bedürfnis nach Systemrückmeldung über das iHMI haben könnte, wurde die Wunschdistanz für eine Systemrückmeldung für drei unterschiedliche interagierende Verkehrsteilnehmende (Erwachsener Fußgänger vs. Fahrradfahrer vs. Kind) untersucht. In Abb. 5 sind die Ergebnisse zur gewünschten mittleren Distanz für eine Systemrückmeldung über das iHMI bei der Annäherung an alle drei Verkehrsteilnehmende dargestellt.

Eine Varianzanalyse mit Greenhouse-Geisser-Korrektur zeigte signifikante Unterschiede für die mittlere gewünschte Distanz einer Systemrückmeldung (*F*(1,82; 191) = 23,37; *p* < 0,001; partielles η^2^ = 0,182). Ein Bonferroni-korrigierter post-hoc Test zur Aufklärung des gefunden großen Effektes zeigte jeweils einen signifikanten Unterschied (beide *p* < 0,001) der gewünschten Distanz für ein Systemfeedback zwischen den Verkehrsteilnehmern Kind und erwachsener Fußgänger (4,14 m; 95 %-CI [2,77; 5,52]) und zwischen dem Kind und dem Fahrradfahrer (2,48 m; 95 %-CI [1,13; 3,83]). Für den Vergleich erwachsener Fußgänger und Fahrradfahrer zeigte sich eine Tendenz, jedoch kein signifikanter Unterschied (*p* = 0,058).

**Figure 6:**
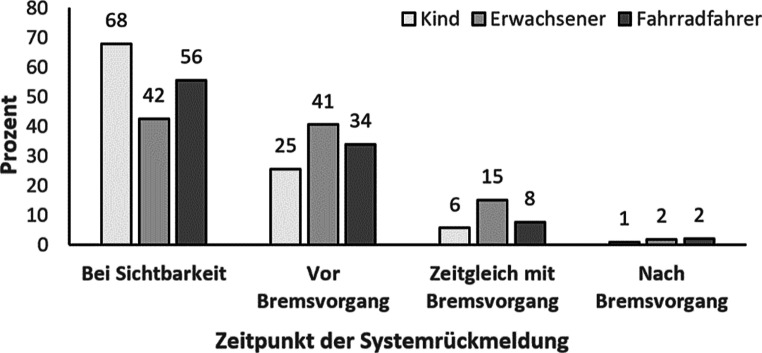

Die subjektiven Aussagen der Versuchsteilnehmenden zum gewünschten Zeitpunkt für eine Systemrückmeldung über das iHMI unterstreichen diesen Befund (Abb. 6). Eine Systemrückmeldung schon beim ersten Auftauchen des Verkehrsteilnehmers wünschten sich 68 % aller Versuchsteilnehmenden, wenn es sich um ein Kind als Interaktionspartner im Verkehr handelte. Auch bei einem Fahrradfahrer wünschten sich bereits 56 % aller Versuchsteilnehmenden eine Systemrückmeldung bei Sichtbarkeit des Verkehrsteilnehmers. Der erwachsene Fußgänger sollte hingegen nur bei 42 % der Versuchsteilnehmenden schon bei Sichtbarkeit angezeigt werden. Unabhängig vom Verkehrsteilnehmer wünschten sich 89 % der Versuchsteilnehmenden eine Systemrückmeldung über das iHMI schon vor dem Bremsvorgang.

**Figure 7:**
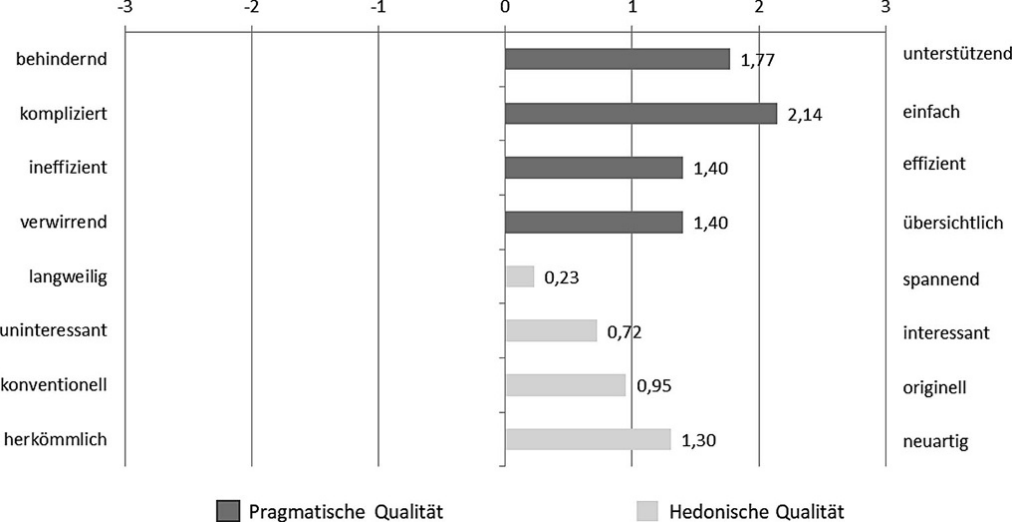

Um die UX des Anzeige- und Interaktionsdesigns des iHMI mit selbstgewähltem Zeitpunkt der Systemrückmeldung zu überprüfen, wurde die Kurzform des UEQ‑S [59] verwendet. Die in Abb. 7 dargestellten Ergebnisse zeigen überdurchschnittlich hohe Ergebnisse in den beiden Subskalen „pragmatische Qualität“ und „hedonische Qualität“. Hierbei wird die einfache, effiziente und unterstützende Komponente des Anzeige- und Interaktionskonzepts des iHMI in seiner pragmatischen Qualität positiv hervorgehoben. Diese Bewertung unterstreicht das Potenzial des vorgestellten iHMI im Sinne eines nutzerfokussierten Designs, dem Bedürfnis von Nutzenden, das Verhalten der Automation im Verkehr zu verstehen, entgegenkommen zu können.

## Diskussion

Im Rahmen dieser Veröffentlichung wurde der Ansatz der nutzerfokussierten Automation eingeführt. Dieser Ansatz propagiert, Systeme so zu gestalten, dass zwei grundlegende, menschliche Bedürfnisse erfüllt werden: das Bedürfnis, verstanden zu werden (BVZW) und das Bedürfnis, zu verstehen (BZV). Der Ansatz verspricht somit, ein adäquates menschliches Vertrauen in automatisierte Systeme zu gewährleisten und die Akzeptanz und damit auch die tatsächliche Nutzung der Systeme zu fördern. Die tatsächliche Nutzung der Systeme entscheidet insbesondere im automobilen Kontext letztendlich über den Erfolg der, z. B. für AVF, in den Markt eingeführten Technologien.

Ebenfalls im Rahmen dieser Veröffentlichung wurde mit Hilfe einer prototypischen Implementierung gezeigt, wie einerseits das BVZW und andererseits das BZV, befriedigt werden kann. Es wurde zunächst erläutert, dass das BVZW befriedigt werden kann, wenn (i) Sensorik im Innenraum des Fahrzeugs relevante Daten (z. B. Sitzposition) über menschliche Zustände (z. B. Aktivität) erfasst und (ii) Algorithmen zur Verfügung stehen, die Informationen über aktuelle Zustände aus den Daten, z. B. mit Verfahren der künstlichen Intelligenz, ableitet. Für die hier berichtete Studie zeigte sich, dass mit diesem Verfahren Stress und Aktivitäten des Nutzenden sehr gut erkannt werden können. Das bedeutet, dass unter Anwendung von Wissen über Nutzungszwecke (z. B. Arbeiten im Mobile Office) und -möglichkeiten (z. B. Entspannen) des Systems sowie Kenntnis von Randbedingungen (z. B. Aufteilung des Innenraums) wesentliche Aspekte der individuellen Erlebenssituation von Nutzenden erfasst und im Rahmen systemsicher Anpassungen auch individuell beantwortet werden kann. Das BVZW kann also mit technischen Mitteln befriedigt werden. Zu berücksichtigen ist jedoch, dass die vorgestellte Zustands- und Aktivitätsmodellierung nur einen eng begrenzten Ausschnitt von möglichen Aktivitäten und -zuständen von Nutzenden umfasst. Die vorgestellte Lösung adressierte insbesondere Stress, der im gewählten Anwendungsfall anhand erfasster Aktivität von Nutzenden als Indikator subjektiver Unsicherheit darüber betrachtet wurde, ob eine wichtige Aufgabe im Mobile Office in der zur Verfügung stehenden Zeit beendet werden kann Die tatsächliche Vielfalt menschlicher Zustände ebenso wie die Zahl und Art zugrundeliegender Faktoren ist dagegen unbegrenzt.

Grundsätzlich stellt sich für zukünftige Forschungsarbeiten die Herausforderung, zu identifizieren, welche Zustände ein technisches System erkennen sollte, damit das BVZW von Nutzenden befriedigt werden kann. Hierbei ist auch zu untersuchen, ob Menschen spezifische Systemeigenschaften zur Erfassung von Nutzenden, die sich auf das BVZW richten, als einen zusätzlichen Nutzen (*perceived usefulness*) betrachten, der auch zu erhöhter Akzeptanz führt. Gleiches gilt für die Bewertung von Funktionen, die sich gezielt auf das BZV richten. Betrachten Menschen sie als eine zusätzliche Erleichterung im Rahmen der Systemnutzung (*perceived ease of use*), und führt dies zu einer erhöhten Akzeptanz? Falls ja, wäre dies in beiden Fällen ein Indikator dafür, dass die TAM-Ansätze auch für Akzeptanzfragen abseits von Arbeitsplätzen berücksichtigt werden können und sollten.

Weiter wurde im Rahmen dieser Publikation erläutert, dass das BZV befriedigt werden kann, indem das iHMI des Fahrzeugs herangezogen wird, um den menschlichen Insassen z. B. zu verdeutlichen, ob Verkehrsteilnehmende erkannt wurden und falls ja, welche dies sind. Der Befund, dass Nutzende intraindividuell unterschiedliche Zeitpunkte für Systemrückmeldungen über das iHMI wünschen, spiegelt die individuell unterschiedlichen Informationsbedürfnisse bei der Nutzung eines AF wieder. Hier fällt besonders auf, dass die Interaktion mit einem Kind – jeweils im Vergleich zu einem Erwachsenen in seinem Verhalten ein eher unberechenbarerer Verkehrsteilnehmer – einen früheren Informationsbedarf über das iHMI vermittelt auslöst. Auch die berichteten Standardabweichungen der jeweiligen Informationswünsche unterstreichen als Streuungsmaß die interindividuellen Unterschiede zwischen den Versuchsteilnehmenden und zeigen die Notwendigkeit für nutzerfokussierte Systemgestaltung zur Vermeidung subjektiver Unsicherheiten auf. Es bestätigt sich also auch in diesem Kontext, dass in Zukunft die Interaktion zwischen einem AF und seinem Passagier individuell gestaltet werden muss, um durch Berücksichtigung individueller Ausprägungen von Ungewissheit und Förderung subjektiver Sicherheit erfolgreich das Vertrauen und die Akzeptanz in die Technologie zu fördern und eine adäquate Nutzung zu provozieren.

Zukünftige Forschung sollte darauf ausgerichtet sein, den hier vorgestellten Ansatz weiter auszuarbeiten. Die hier vorgestellte Studie zur Entwicklung von unsicherheitsreduzierenden iHMI-Lösungen arbeitete mit Online-Medien, die einerseits eine große Datenmenge und somit eine hohe statistische Validität erfüllen, andererseits aufgrund der mangelhaften externen Validität jedoch kritisiert werden können. Eine weitere Herausforderung wird außerdem sein, den Ansatz der nutzerfokussierten Automation auf mehrere Interaktionspartner zu erweitern. Die meisten Fahrzeugkonzepte berücksichtigen die Nutzung durch mehrere Insassen. Ist es möglich, bei jedem Passagier die beiden Bedürfnisse BZV und BVZW zu stillen? Und wie verhält es sich im externen Umfeld des Fahrzeugs? Auch hier ist das Fahrzeug i. d. R. nicht nur mit einem Gegenüber, sondern mit verschiedensten Verkehrsteilnehmenden konfrontiert. Es gilt also, Algorithmen zu entwickeln, die auch auf die Distanz hin in der Lage sind, relevante Zustände anderer Verkehrsteilnehmer zu erfassen und valide zu diagnostizieren. Nicht zuletzt sollten Interaktionsparadigmen entwickelt werden, die das BZV anderer Verkehrsteilnehmer befriedigen. Hierbei ist es besonders wichtig, dass entsprechend konsistent mit allen menschlichen Interaktionspartnern innerhalb und außerhalb des Fahrzeugs kommuniziert wird. Wenn diese Herausforderungen gemeistert sind, dann ist ein sehr großer Schritt in die Richtung der Umsetzung nutzerfokussierter Automation und der Gewährleistung subjektiver Sicherheiten getan, so dass diese Automationssysteme akzeptiert und tatsächlich genutzt werden.

## Footnotes

1 Es ist möglich, dass die gewählten Aufgaben vermutlich nicht bei allen Versuchsteilnehmenden das gleiche Stresslevel erzeugt haben, da Stress von den eigenen erlebten Anpassungsfähigkeiten abhängt (vgl. [48]). Somit könnte eine Optimierung der Aufgabe mit einer Anpassung an die persönlichen Fähigkeiten der Versuchsteilnehmenden zu einer verbesserten Induktion von Stress führen.
